# Effects of Chronic and Acute Intraocular Pressure Elevation on Scotopic and Photopic Contrast Sensitivity in Mice

**DOI:** 10.1167/iovs.16-19312

**Published:** 2016-06-10

**Authors:** Meike E. van der Heijden, Priya Shah, Cameron S. Cowan, Zhuo Yang, Samuel M. Wu, Benjamin J. Frankfort

**Affiliations:** 1 Department of Ophthalmology, Baylor College of Medicine, Houston, Texas, United States; 2 Department of Neuroscience, Baylor College of Medicine, Houston, Texas, United States

**Keywords:** glaucoma, intraocular pressure, contrast sensitivity

## Abstract

**Purpose:**

To compare the impact of intraocular pressure (IOP) elevation on scotopic and photopic contrast sensitivity in mice.

**Methods:**

We chronically elevated the IOP of wild-type mice via injection of polystyrene beads or acutely via injection of highly cohesive sodium hyaluronate. Some eyes with chronically elevated IOP were treated with either topical brimonidine tartrate 0.1% or brinzolamide 1%. Scotopic and photopic contrast sensitivity was assessed at peak spatiotemporal frequencies at multiple time points, with an established optokinetic technique. Retinal ganglion cell (RGC) counts were determined with an antibody to class III beta-tubulin. Correlations among IOP level, RGC count, and scotopic or photopic contrast sensitivity were performed.

**Results:**

Six weeks of IOP elevation caused a generalized reduction of photopic contrast sensitivity and a preferential reduction of scotopic contrast sensitivity at peak spatiotemporal frequencies. The administration of brinzolamide but not brimonidine caused a significant reduction in cumulative IOP, whereas brimonidine, but not brinzolamide, prevented RGC loss. Both brimonidine and brinzolamide prevented contrast sensitivity loss, but brimonidine did so at earlier time points and across a wider range of lighting conditions. Following either chronic or acute IOP elevation, scotopic contrast sensitivity was impacted most prominently by IOP level and not by RGC count, while photopic contrast sensitivity was impacted by a combination of factors.

**Conclusions:**

It is possible that scotopic-specific retinal circuitry is altered preferentially by IOP elevation, and that changes in scotopic contrast sensitivity will assist with glaucoma detection. Brimonidine appears to prevent RGC loss via an IOP-independent mechanism.

Glaucoma is a leading cause of blindness worldwide and a major public health concern.^1,2^ The disease is characterized by loss of the output cells of the retina, the retinal ganglion cells (RGCs), which leads to worsening of vision over time. At this time, the only conclusively identified modifiable risk factor for glaucoma is intraocular pressure (IOP), and essentially all treatments for glaucoma—pharmacologic, laser, and surgical—are based on the reduction of IOP.^3,4^ While IOP-lowering medications are the mainstay of therapy, there have been only a small number of prospective studies to demonstrate that these drugs preserve visual function in glaucoma patients. Furthermore, several recently developed agents have gained acceptance into clinical practice based on the assumption that the reduction of IOP by any method leads to the preservation of vision. However, one recent randomized controlled trial calls this assumption into question, showing that while two common IOP-lowering medications were equally effective at IOP lowering, their impact on visual preservation was quite different.^5^ These results hint at a more complex relationship between IOP and visual function than previously assumed.

Other clinical data suggest that contrast sensitivity may be among the earliest aspects of visual function to be impacted in patients with glaucoma.^6,7^ In addition, scotopic (dark, or rod-based) visual function has been shown to be disrupted in clinical and experimental settings. First, patients with glaucoma are known to have poor vision in dark conditions and during transitions between dark and light conditions.^8–11^ Second, evidence from animal models of glaucoma suggests that scotopic visual function, on both the retinal and cellular level, is rapidly impacted by elevations in IOP.^12–15^ Since the scotopic and photopic (light, or cone-based) visual pathways incorporate distinct retinal circuitry, it is possible that IOP elevation may affect scotopic and photopic contrast sensitivity differently, and that loss of scotopic contrast sensitivity might be an early indication of disease.

In this manuscript, we report the first direct comparisons between scotopic and photopic visual function under conditions of experimental glaucoma in mice. To do so, we assessed contrast sensitivity using optokinetic reflexes (OKRs) under both lighting conditions in animals exposed to elevated IOP induced by one of two experimental glaucoma models. To generate mild, chronic increases in IOP, we used a variation of the well-established “microbead occlusion” model, which involves the injection of micron-range diameter beads into the anterior chamber to occlude the trabecular meshwork and cause a secondary elevation of IOP.^12,16–18^ Furthermore, we applied IOP-lowering drops with distinct biological mechanisms in conjunction with this model to separate out the effects of RGC loss from RGC dysfunction. To generate high, acute increases in IOP, we used a new model of unilateral sodium hyaluronate injection. With both models, we found that scotopic contrast sensitivity, rather than photopic contrast sensitivity, is preferentially impacted by IOP elevation. These comparisons highlight potential changes to scotopic-specific retinal circuitry caused by IOP elevation, and suggest a possible role for scotopic contrast sensitivity in glaucoma diagnosis.

## Materials and Methods

### Animals

We purchased 5-week-old female *C57BL*/*6* mice from Jackson Laboratories (Bar Harbor, ME, USA), acclimated for 1 week prior to experimentation. All animals were treated in accordance with National Institutes of Health (NIH) guidelines, the ARVO Statement for the Use of Animals in Ophthalmic and Vision Research, and the Baylor College of Medicine Institutional Animal Care and Use Committee welfare guidelines.

### Intraocular Pressure Elevation

To achieve a chronic elevation of IOP, mice were first anesthetized with a weight-based intraperitoneal injection of ketamine, xylazine, and acepromazine. One eye was dilated with 1% tropicamide. Additional topical anesthesia was provided with a drop of 0.5% proparacaine. Anterior chamber injection of polystyrene beads into the dilated eye was performed as described previously.^12,19^ Following bead injection, a drop of 0.5% moxifloxacin was placed on the cornea. The other eye was not injected and served as an intra-animal control. Mice that were treated with IOP-lowering medications received a single drop of either brimonidine tartrate 0.1% (Alphagan P; Allergan, Inc., Dublin, Ireland) or brinzolamide 1% (Azopt; Alcon, Fort Worth, TX, USA) once per day for the duration of the experiment, including weekends. This drop was administered within a strict time window: between 10 AM and 2 PM.

For the acute elevation of IOP, we performed an injection of highly cohesive sodium hyaluronate (Healon 5, Abbott Medical Optics, Abbott Park, IL, USA) into the anterior chamber of one eye, while the other eye served as an uninjected, intra-animal control. Animals were anesthetized with inhaled isoflurane that was administered through a nose cone. Additional topical anesthesia was provided with a drop of 0.5% proparacaine. The midperipheral cornea of one eye was punctured with a fresh 30-gauge needle, and a 75-μm diameter pulled glass micropipette attached to a tube of sodium hyaluronate was inserted through the cornea track incision. Sodium hyaluronate was injected to fill the anterior chamber and the IOP measured to ensure an elevation to at least 35 mm Hg. After the procedure, a drop of 0.5% moxifloxacin was placed on the cornea of injected eyes. The procedure was repeated on the same eye weekly for four cycles. For subsequent IOP elevations, the same eye was injected at different locations on the cornea.

The IOP of all eyes was measured using a rebound tonometer calibrated for mouse use as previously described.^12^ Intraocular pressure was measured according to a precise schedule and at the same time of day. For chronic IOP elevation, the IOP was measured prior to bead injection, and then twice per week for 6 weeks. For acute IOP elevation, the IOP was measured just prior to sodium hyaluronate injection and then immediately after injection. Subsequent IOP measurements were taken at postinjection days 1 and 6. The cycle was then repeated four times as described above.

### Measurement of OKRs

Animals were dark-adapted for at least 2 hours prior to testing. Baseline photopic and scotopic contrast sensitivities were tested prior to initial injection via either method with an established OKR-based technique.^20^ Scotopic contrast sensitivities were determined at least 1 hour prior to photopic testing. We repeated OKR experiments according to a precise schedule, depending on the mechanism of IOP elevation. For chronic IOP elevation, contrast sensitivity was measured every 2 weeks (postinjection weeks 2, 4, and 6). For acute IOP elevation, contrast sensitivity was measured immediately after dark adaptation following sodium hyaluronate injection, and then again on postinjection days 1 and 6 (which is the same as the day prior to injection for the subsequent week of the cycle).

We tested OKRs using a custom-built apparatus.^20^ Mice were placed on an elevated platform inside a box of four computer screens that display a virtual cylinder with moving vertical gratings at a range of spatiotemporal frequencies, similar to other techniques.^21–26^ The bottom and top of the box were mirrored to give the illusion of infinitely tall vertical gratings. In the center of the upper mirror, directly above the platform, a camera was positioned, to allow a trained observer to track to the mouse's movements. An infrared filter in front of the camera prevented the observer from seeing the stimulus presented on the screens. The contrasts of the presented gratings were controlled by a custom protocol written in MATLAB. The protocol was a slope-constrained variant of the ψ method, a Bayesian adaptive approach for estimating the mouse's psychometric function for contrast detection.^27^ The contrast of the subsequent trial is chosen to maximize its expected information by using a 1-step-ahead search. The threshold was determined to be the contrast that evoked correct responses of the animal half of the time. Contrast sensitivity was then defined as the inverse of percent contrast threshold. All experiments started with a prior that was based on extended previous testing of *C57BL*/*6* mice.^20^ The mean photopic light intensity was 0.87 log_10_ cd/m^2^ (1.93 log_10_ photoisomerizations/rod). Light intensity was attenuated to the scotopic range by placing neutral density filters in front of the screens, thereby decreasing mean light intensity to −2.3 log_10_ cd/m^2^ (−1.08 log_10_ photoisomerizations/rod).^20^ Relative intensities ranged approximately 2 log units from peak to peak at highest contrast.

Experiments consisted of 100 stimuli, randomly drawn from a pool of 50 leftward and 50 rightward moving gradients. Mice respond only to stimuli moving in the temporal to nasal direction, and following each stimulus, the trained observer—who was masked to the direction of the stimulus—chose the expected direction of the stimulus based on the mouse's head movement according to a two-alternative forced choice paradigm.^20,23,28^ During the entire experiment, the observer was also masked to which eye was exposed to IOP elevation to minimize bias.

### Immunohistochemistry and Cell Counting

Retinas were dissected, whole mounted, and fixed as previously described.^12^ Retinas were incubated in primary antibody against the RGC-specific marker, class III beta-tubulin (TUJ1, 1:500; Covance, Princeton, NJ, USA).^29^ After washing, secondary antibody (donkey-mouse 488, 1:300) and a fluorescent nuclear dye (TO-PRO3, 1:1000; Molecular Probes, Eugene, OR, USA) were added for counterstaining. Retinas were then washed and mounted in medium (Vectashield; Vector Laboratories, Burlingame, CA, USA).

Retinas were imaged with a laser confocal microscope (LSM 510; Leica Microsystems, Wetzlar, Germany) and images were processed with commercial software (Zeiss LSM-PC; Carl Zeiss Microscopy, Jena, Germany). Retinal images for cell counting were obtained as previously described.^12^ Tubulin-positive marked cells were manually counted by a single masked observer, assisted by ImageJ software (http://imagej.nih.gov/ij/; provided in the public domain by the National Institutes of Health, Bethesda, MD, USA). These numbers were used to convert the cell counts into cells/mm^2^. A second masked observer recounted several regions to verify the results.

### Statistical Analysis

All analysis was performed using statistical software (SPSS Statistics Version 21; IBM Corp., Armonk, NY, USA). Values in text and figures are presented as averages ± SEM and a *P*-value < 0.05 was considered as statistically significant. We used ANOVAs to compare the difference between different groups and repeated measured ANOVAs to compare between groups at different times. When there was a significant effect on the group level, additional Bonferroni post hoc analyses were performed to detect pairwise differences between groups. Cumulative IOP difference was calculated as the sum of the differences in IOP between uninjected and injected eyes of the same animal. Similarly, log contrast sensitivity loss was calculated as the difference between uninjected and injected eyes of the same animal.

## Results

**Figure 1:**
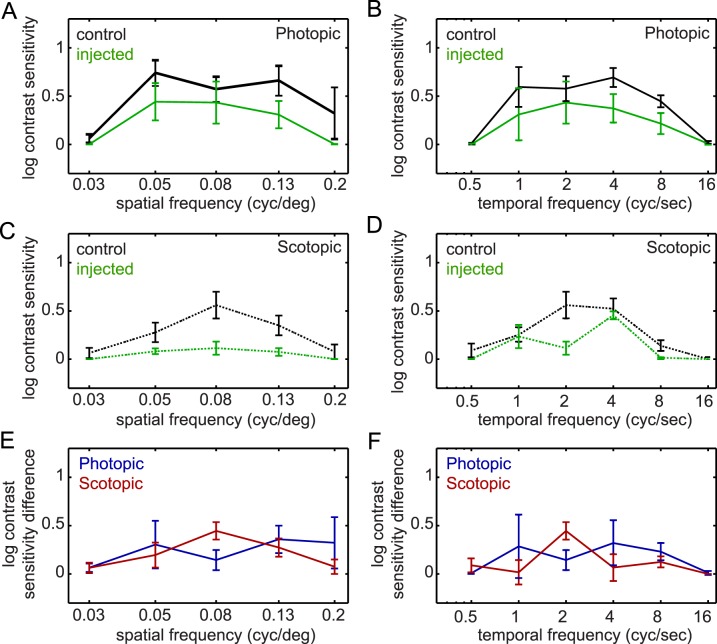
Spatiotemporal tuning of contrast sensitivity after bead injection. Contrast sensitivity was tested 6 weeks after bead injection over a range of spatial frequencies with constant temporal frequency (temporal frequency, 2 cyc/sec; [**A**, **C**, **E**]) or over a range of temporal frequencies with constant spatial frequency (spatial frequency, 0.08 cyc/deg; [**B**, **D**, **F**]). (**A**–**D**) Contrast sensitivity for all control (uninjected) eyes was tuned to spatial and temporal frequency in a bandpass manner, under both photopic ([**A**, **B**] *black solid lines*) and scotopic ([**C**, **D**] *black dotted lines*) lighting conditions (*P* < 0.001 for all). Compared with these uninjected eyes, contrast sensitivity of contralateral bead-injected eyes (*solid* and *dotted green lines* [**A**–**D**]) was statistically decreased under both lighting conditions (photopic, *P* = 0.03; scotopic, *P* < 0.001). (**E**, **F**) Losses in contrast sensitivity between the uninjected and injected eyes of the same animal were calculated to determine if alterations in spatiotemporal tuning occurred following bead injection. No differences in spatiotemporal tuning between injected and uninjected eyes were found under photopic conditions (*blue lines*). However, scotopic tuning was affected by bead injection (*red lines*; *P* < 0.001 for both spatial [**E**] and temporal [**F**] conditions). Scotopic contrast sensitivity was differentially affected at spatiotemporal frequencies that elicited the most sensitive responses in uninjected eyes (peak spatiotemporal frequencies; spatial frequency, 0.08 cyc/deg; temporal frequency, 2 cyc/sec), whereas losses in photopic contrast sensitivity were statistically equal over the tested range (*n* = both eyes of 4 animals per group; mean ± SEM).

The measurement of optokinetic responses (OKRs) can be used in mice to accurately detect changes in visual function that occur following various experimental interventions, including IOP elevation.^21–26,28,30–33^ Since contrast sensitivity may be disrupted in patients with early stage glaucoma, we chose to initially study contrast sensitivity in mice in which IOP was chronically elevated to mild levels (uninjected eyes: 10.1 ± 0.3 mm Hg; injected eyes: 12.1 ± 1.2 mm Hg). In wild-type mice, OKRs occur in response to a wide range of spatiotemporal information and exhibit high sensitivity to moderate values, but low sensitivity to extreme highs or lows, with responses degrading gradually as these extremes are approached.^20,24,25^ To determine whether contrast sensitivity was lost uniformly across the spatiotemporal frequency spectrum, four mice with elevated IOP were tested over a wide range of spatiotemporal frequencies. Following 6 weeks of IOP elevation, we found that the expected band-pass pattern of contrast sensitivity was present (lowest at minimal and maximal spatial and temporal frequencies, highest in between), and that contrast sensitivity was consistently reduced at all tested spatial and temporal frequencies under both photopic and scotopic conditions (Figs. 1A–D).

However, the patterns of contrast sensitivity reduction differed with lighting condition. Under photopic conditions, neither the spatial (*P* = 0.51) nor temporal (*P* = 0.07) frequency impacted the reduction in contrast sensitivity (Figs. 1E–F, blue lines), whereas under scotopic conditions, the reduction in contrast sensitivity was highest at peak spatial and temporal frequencies (*P* < 0.001 for both; Figs. 1E–F, red lines). Thus, we found that contrast sensitivity was not only highest at peak spatiotemporal frequencies, but might also be most affected at these frequencies. We therefore tested animals at peak spatiotemporal frequencies (spatial frequency = 0.08 cyc/deg; temporal frequency = 2 cyc/sec) for all additional experiments.

**Figure 2:**
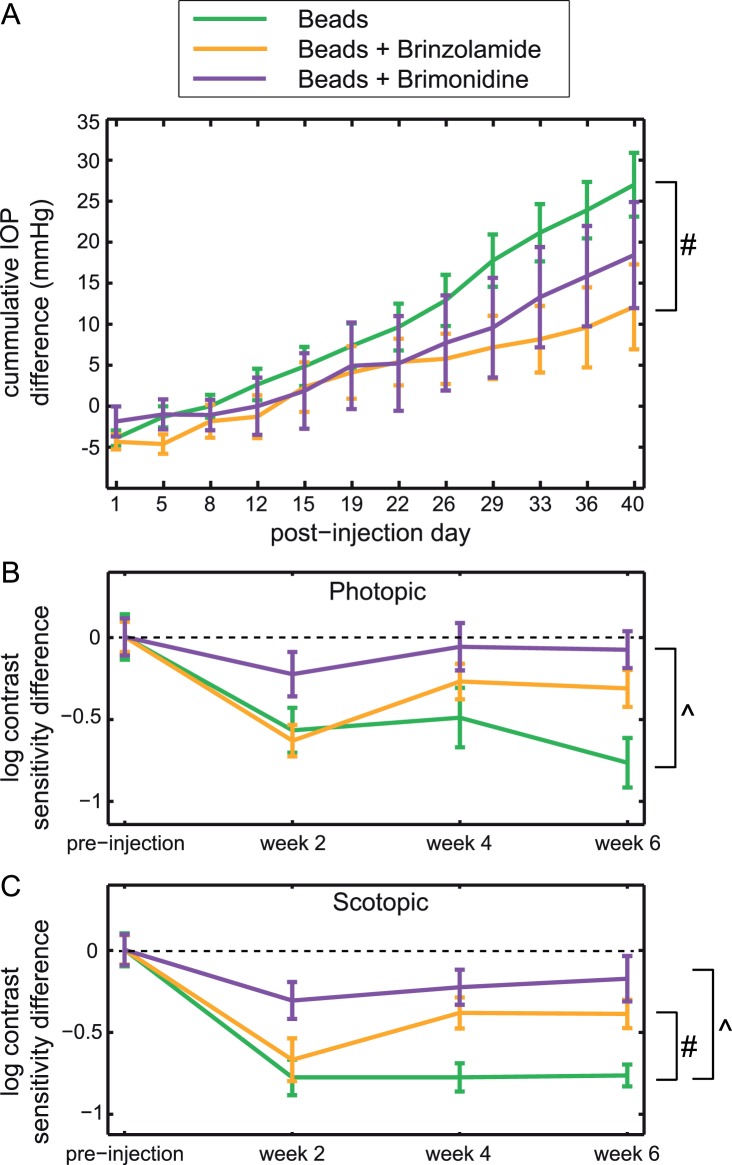
Cumulative IOP difference and peak contrast sensitivity loss after bead injection. (**A**) Cumulative IOP difference of bead-injected eyes was increased across the entire range of the study (beads, *green*) and was attenuated by both brinzolamide (*orange*) and brimonidine (*purple*). A post hoc analysis revealed that only brinzolamide-treated animals had a statistically significant decrease in cumulative IOP difference compared with animals that did not receive IOP-lowering drops (1-way ANOVA, Bonferroni post hoc; *P* = 0.041; *bracket with #*). While brimonidine treatment trended toward lowering IOP, this effect was not significant compared with animals that did not receive IOP-lowering drops (Bonferroni post hoc: *P* = 0.184; *n* = 17 for beads only, 9 for brinzolamide, and 12 for brimonidine). (**B**, **C**) Contrast sensitivity loss was observed under both photopic (**B**) and scotopic (**C**) conditions in animals that received a unilateral bead injection but not IOP-lowering eye drops (*green*). While brinzolamide treatment (*orange*) attenuated this contrast sensitivity loss in both lighting conditions, it only resulted in a significant difference compared with animals that did not receive treatment in scotopic conditions (ANOVA, Bonferroni post hoc: photopic, *P* = 0.159; scotopic, *P* = 0.002; *bracket with #*). Brimonidine (*purple*) attenuated the contrast sensitivity loss even further and resulted in contrast sensitivity loss that was statistically smaller than the loss observed in bead-treated animals in both light intensities (ANOVA, Bonferroni post hoc: *P* < 0.001 for both photopic and scotopic; *brackets with ^*; none, *n* = 7; brinzolamide, *n* = 9; brimonidine, *n* = 7). Mean ± SEM is shown for all panels.

While it is well established that anterior chamber bead injections result in elevation of IOP in mice, few studies confirm that IOP-lowering medications work to lower IOP and preserve anatomy after bead injection.^34^ Furthermore, no studies have assessed visual function after lowering the IOP of bead-injected eyes. Since daily administration of both brimonidine and brinzolamide has been confirmed to reduce IOP in a similar bead injection model, we first confirmed their abilities to lower IOP in our model. Both agents blunted the cumulative IOP increase seen from bead injection, but neither eliminated it. Over the course of the 6-week study, the mean daily IOP percentage increases (±SEM) in eyes that received only bead injection, bead injection + daily brimonidine, and bead injection + daily brinzolamide were 26.9% ± 2.3%, 21.9% ± 3.0%, and 20.0% ± 2.6%, respectively. Viewed as a cumulative IOP difference over time, this effect was statistically significant only for brinzolamide (Fig. 2A).

To determine if the reduction in IOP caused by brimonidine and brinzolamide also preserved visual function, we compared the OKRs at peak spatiotemporal frequencies of bead-injected eyes to those of bead-injected eyes treated daily with either IOP-lowering agent. At each time point (baseline and 2, 4, and 6 weeks post injection), we calculated the intra-animal difference in contrast sensitivity between the treated and untreated eye to determine the contrast sensitivity difference (Figs. 2B, 2C). Bead-injected eyes showed marked contrast sensitivity loss under both photopic and scotopic conditions by 2 weeks after injection, which remained constant through the rest of the experiment (*P* < 0.001). Interestingly, both brinzolamide and brimonidine mitigated this contrast sensitivity loss, yet did so in statistically distinct manners. Under photopic conditions, brimonidine, but not brinzolamide, caused an attenuation of contrast sensitivity loss that was statistically significant (ANOVA with repeated measures, Bonferroni post hoc: brinzolamide, *P* = 0.159; brimonidine, *P* < 0.001). Under scotopic conditions, however, both brinzolamide and brimonidine protected against contrast sensitivity loss (ANOVA with repeated measures, Bonferroni post hoc: brinzolamide, *P* = 0.002; brimonidine, *P* < 0.001). Under both photopic and scotopic conditions, there was minimal effect of brinzolamide at the earliest time point (2 weeks), but moderate and similar effectiveness at later time points (4 and 6 weeks). Taken together, these data suggest that both brinzolamide and brimonidine can preserve vision in vivo by preventing the contrast sensitivity loss seen following IOP elevation via bead injection, but the pattern of this preservation is different and may occur via distinct mechanisms.

**Figure 3:**
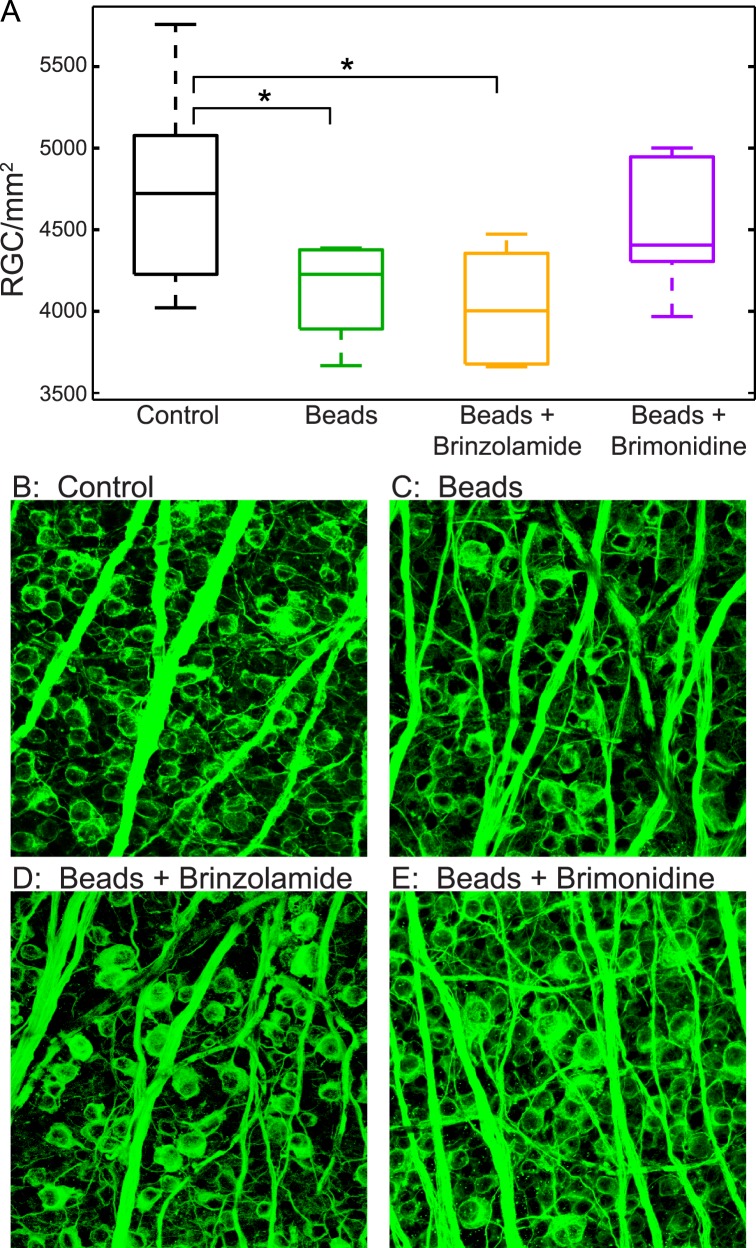
Retinal ganglion cell numbers after bead injection. (**A**) *Box-and-whisker* plots showing RGC number in flatmounted retinas. Eyes injected with beads had a statistically significant smaller number of RGCs compared with control (uninjected) eyes. * Bonferroni post hoc: *P* = 0.045 (*green*; *n* = 7 eyes). A similar loss of RGCs was observed in eyes injected with beads that received treatment with brinzolamide. * Bonferroni post hoc: *P* = 0.031 (*orange*; *n* = 6 eyes), but was not observed in injected eyes were treated with brimonidine (Bonferroni post hoc: *P* = 1; *purple*; *n* = 7 eyes). All post hoc analyses were performed where injected eyes were compared with RGC number in uninjected control eyes (*black*, *n* = 13 eyes). (**B**–**E**) Representative retinal flatmount images of class III beta-tubulin staining (*green*).

Next, we sought to compare the anatomic impact of IOP reduction by brinzolamide and brimonidine after bead injection. Thus, at the end of the 6-week study period, after all OKR measurements, we assessed the number of RGCs present in retinal flat mounts with a well-established antibody to class III beta-tubulin, which is thought to label all RGCs (Fig. 3).^29^ Interestingly, we found that bead-injected eyes, and bead-injected eyes treated with brinzolamide, showed a reduction in RGC count from uninjected control eyes (control, 4767 ± 187 RGCs/mm^2^ [mean ± SEM]; beads, 4138 ± 232 RGCs/mm^2^; beads + brinzolamide, 4105 ± 131 RGCs/mm^2^; *P* < 0.05 for beads and beads + brinzolamide when compared with control). Conversely, no reduction in RGC count was seen in bead-injected eyes treated with brimonidine, suggesting that daily brimonidine treatment prevented IOP-mediated RGC loss, whereas brinzolamide did not (beads + brimonidine, 4547 ± 166 RGCs/mm^2^; *P* > 0.05 when compared with control). Interestingly, as brimonidine was less effective at lowering IOP than brinzolamide, this suggests that RGCs may be preserved in these eyes by an IOP-independent mechanism.

**Figure 4:**
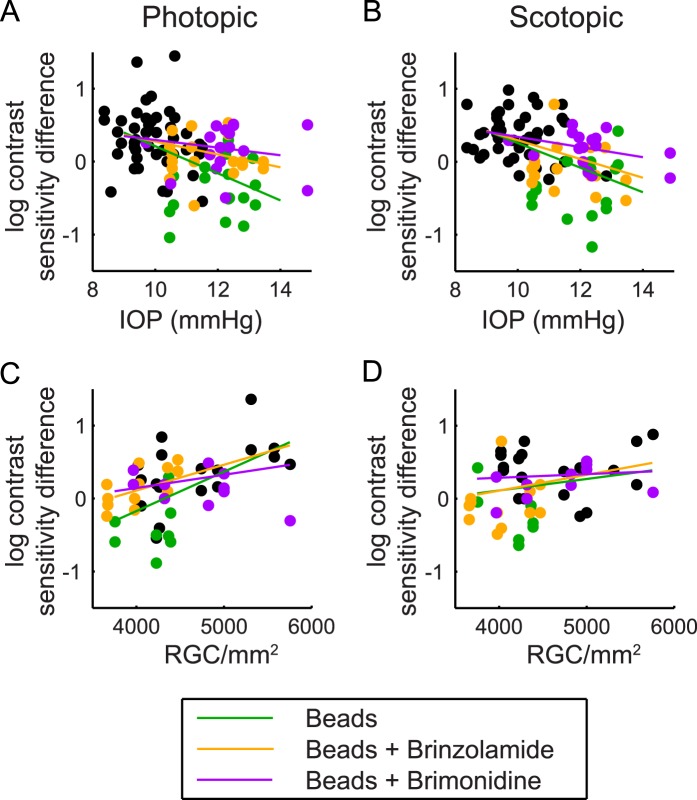
Correlations with contrast sensitivity difference. Individual data points (*dots*) were collected from uninjected eyes (*black*); bead-injected eyes (*green*); bead + brinzolamide eyes (*orange*); and bead + brimonidine eyes (*purple*). Depicted slopes are analyzed as the correlation of the *x* and *y* variable for all uninjected eyes + the injected eyes of the respective group (beads, *green*; beads + brinzolamide, *orange*; beads + brimonidine, *purple*). Correlations were performed for mean IOP and contrast sensitivity difference ([**A**] photopic; [**B**] scotopic) and mean RGC count and contrast sensitivity difference ([**C**] photopic; [**D**] scotopic). Under scotopic conditions, there are consistently correlations between IOP level and contrast sensitivity, but not between RGC count and contrast sensitivity. Under photopic conditions, correlations are present between both IOP level and RGC count with contrast sensitivity, except when treated with brimonidine (*R*^2^ values, slopes, and *P* values are reported in the Table).

We then tried to further explain the relationship between IOP, RGC count, and contrast sensitivity difference. To do so, we calculated the mean photopic and scotopic contrast sensitivity difference for all eyes (contrast sensitivity after 6 weeks minus contrast sensitivity prior to bead injection), and plotted these values against either the average IOP (average of the 12 time points post injection) or the RGC count. We then determined whether these relationships were changed by the use of drops by populating the correlation with all control eyes and the respective data points from each group. We found very different results for scotopic and photopic contrast sensitivity. Under scotopic conditions, there was a consistent and strong correlation with IOP, as well as a consistent absence of a relationship with RGC count, regardless of treatment group. These findings suggest that scotopic contrast sensitivity is impacted most prominently by IOP level and not by RGC count. Under photopic conditions, however, correlations were present for both IOP and RGC count, but only for bead-injected eyes and bead-injected eyes treated with brinzolamide. These data suggest a more complex relationship among IOP, RGC count, and contrast sensitivity under photopic conditions that is altered by the use of brimonidine but not brinzolamide (Fig. 4; Table).

**Table:**
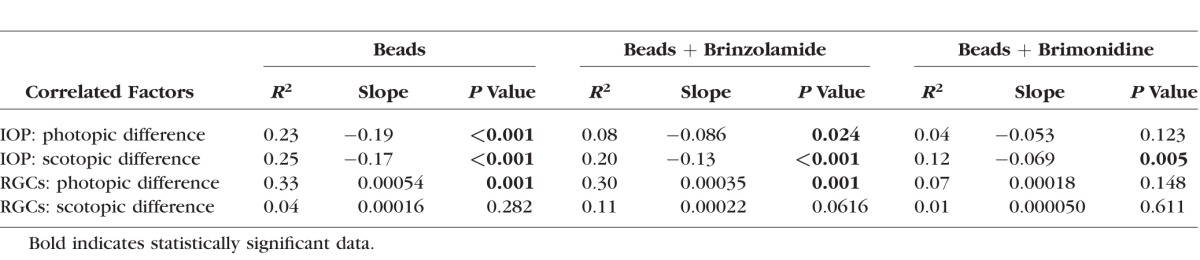
Correlation Coefficients, Slopes, and *P* Values

**Figure 5:**
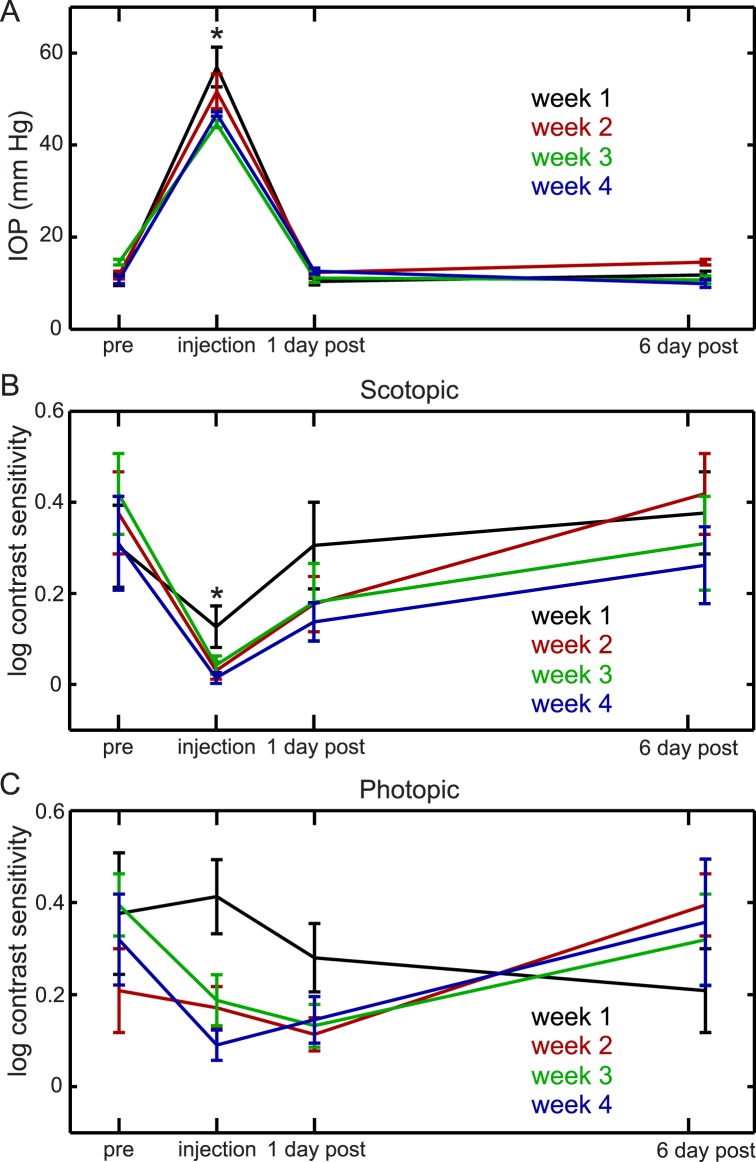
Peak contrast sensitivity after sodium hyaluronate injection. Intraocular pressure in injected eyes was measured on the day before IOP elevation (pre), immediately after injection (injection), and on the day following elevation (1 day post) for four weekly cycles (weeks 1–4). Log contrast sensitivity of injected eyes at peak spatiotemporal frequencies was measured on the day before IOP elevation (pre), 2 hours after injection (injection), and on the day following elevation (1 day post) for four weekly cycles (weeks 1–4). The label “6 days post” for each week is the same measurement as “pre” for the following week. (**A**) Mean IOP; IOP was only elevated immediately after sodium hyaluronate injection (*asterisk*). (**B**) Mean log scotopic contrast sensitivity. Scotopic contrast sensitivity loss occurred uniformly at each week of treatment, with a single reduction on the day of injection. *P = 0.001, all weeks. (**C**) Mean log photopic contrast sensitivity. Photopic contrast sensitivity loss was less during the first week of treatment (week 1, *black*) than in the following weeks (all other colors; repeated measures ANOVA, *P* = 0.02) and did not follow a specific pattern within the weeklong injection cycle (*n* = 8 for all panels). Mean ± SEM is shown for all panels.

Finally, to further explore our observation that IOP level had a stronger effect on scotopic than photopic contrast sensitivity, we developed an alternative model of IOP elevation based on anterior chamber injection of highly cohesive sodium hyaluronate to acutely increase IOP. With this model, we were able to directly test the hypothesis that acute increases in IOP preferentially impact scotopic contrast sensitivity, because IOP became elevated shortly after injection and then rapidly recovered to and remained at baseline pressures (Fig. 5A). We measured contrast sensitivity prior to, immediately after, and 1 and 6 days after IOP elevation to determine acute and prolonged effects, and repeated this cycle of IOP elevation and contrast sensitivity measurement four times to determine any additive effects. We found that both photopic and scotopic contrast sensitivity were diminished immediately after IOP elevation (testing 2 hours after injection, 3-way ANOVA [day of experiment × week of repetition × lighting condition]: effect of day on contrast sensitivity: *P* < 0.001, Bonferroni post hoc, injection day is different from preinjection day and postinjection day: *P* < 0.001 and *P* = 0.001, respectively), but that the reduction in contrast sensitivity was more profound and more temporally linked with acute IOP elevation under scotopic than photopic conditions (Figs. 5B, 5C). Furthermore, scotopic contrast sensitivity also recovered rapidly to baseline levels once IOP normalized, whereas photopic contrast sensitivity did not (interaction between day of experiment × lighting condition: *P* = 0.004). These repeated, acute increases in IOP did not affect postmortem RGC counts (class III beta-tubulin positive cells: 4604 ± 72 cells/mm^2^ for injected eyes versus 4644 ± 59 cells/mm^2^ for control eyes), suggesting that scotopic contrast sensitivity loss occurs independently of RGC number. These data support our findings following chronic IOP elevation, namely that scotopic visual impairment may be directly affected by IOP level and is independent of RGC number, and that photopic visual impairment may be mediated by a more complex relationship among several factors.

## Discussion

In this study, we used a modified version of an established OKR-based technique to estimate both photopic and scotopic contrast sensitivity in mice after IOP elevation. Optokinetic reflex–based testing has been used previously to distinguish photopic and scotopic phenotypes.^20,22,25^ However, the use of OKR-based testing in animal models of glaucoma has been limited to chronic models and has focused exclusively on photopic measurements.^30,31,35,36^ This is the first work to combine a detailed assessment of photopic and scotopic contrast sensitivity phenotypes with experimental models of IOP elevation in mice.

**Figure 6:**
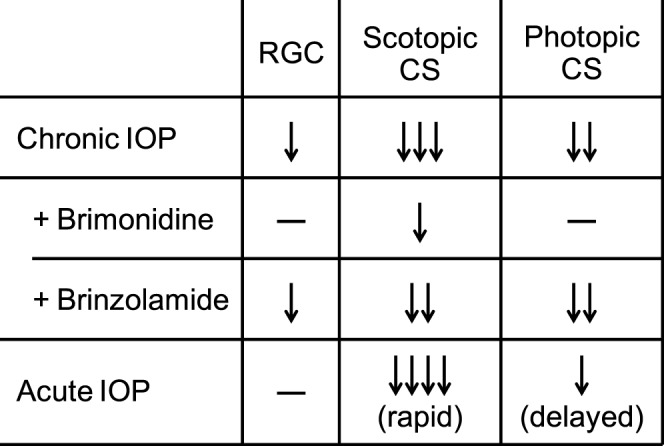
Summary of experimental data.

We elevated IOP by two distinct methods. First, we used a microbead-based model to induce chronic IOP elevation, and paired this approach with IOP-lowering medications to probe the impact of IOP and RGC count on contrast sensitivity. Second, we used a novel sodium hyaluronate injection model to induce acute IOP elevation and confirm the impact of IOP level on contrast sensitivity. Our findings with both models suggest that scotopic and photopic contrast sensitivities are altered differently in response to IOP elevation, and that scotopic contrast sensitivity is more directly impacted by IOP level and potentially independent of RGC number (Fig. 6). This distinction further highlights the importance of the systematic dissection of the visual circuitry to accurately study glaucoma in experimental systems.

Contrast sensitivity loss in response to chronic IOP elevation followed a specific pattern according to the lighting condition. Under photopic conditions, contrast sensitivity decreased in a manner which was independent of spatiotemporal tuning. That is, contrast sensitivity loss occurred equally at all tested spatial and temporal frequencies. Under scotopic conditions, contrast sensitivity loss also occurred across all tested spatial and temporal frequencies, but the magnitude of loss was greatest at peak frequencies. This is consistent with prior observations that RGCs have different spatial and temporal preferences under different lighting conditions.^37–39^ It is possible that IOP elevation could alter these spatiotemporal preferences, potentially via disruption of an amacrine cell mediated circuit.^13^ Furthermore, the observed loss of peak contrast sensitivity under scotopic conditions could potentially explain the common complaint that patients with glaucoma have difficulty seeing in dark conditions and when adjusting to rapid changes in ambient light.^8–11^

We also found that brinzolamide and brimonidine are both able to lower IOP, but only brimonidine prevented IOP-induced RGC loss. This discrepancy suggests that brimonidine has an additional, IOP-independent, and possibly neuroprotective mechanism, which has been postulated in both animal and clinical studies.^5,40,41^ This preservation of RGCs in mice treated with brimonidine allowed us to determine that scotopic contrast sensitivity changed in response to IOP level itself, whereas photopic contrast sensitivity changed as a consequence of IOP and additional factors, such as RGC count. These findings were supported by measurements made after acute IOP elevation, in which we detected a severe reduction of scotopic contrast sensitivity that recovered as IOP normalized. Since scotopic contrast sensitivity relies on a complex relationship of RGCs and various retinal interneurons, in particular AII amacrine cells (AIIACs), these findings suggest that IOP may have preferential effects on specific connections in the retina, such as those mediated by amacrine cells that are critical for rod pathway–mediated visual function.^42,43^ Previous work with single cell electrophysiology and transgenic animals in models of experimental glaucoma have already implicated AIIACs as a critically affected cell type, and our data strongly support this relationship, which appears to exist at both chemical synapses and connexin-mediated gap junctions.^13,20^ This is also consistent with a recent ERG study in rats that found that the scotopic ERG was more sensitive to IOP elevation than the photopic ERG,^44^ as well as additional ERG studies in rodents that detected abnormal scotopic responses in the setting of elevated IOP.^12,15,45–48^ Another possible explanation is that direction-selective retinal ganglion cells (DSGCs), which are critical for the contrast-dependent OKR and can be impacted by IOP elevation, display variable susceptibility to IOP elevations depending on their adaptive state.^49–51^ That is, DSGCs function relatively normally under conditions of high IOP when light-adapted, but poorly when dark-adapted. Finally, there is evidence for RGC subtype-specific IOP related phenotypes, as well as indirect evidence that RGC light sensitivity can change according to adaptive state.^31,36,39,51^ As these subtype differences include differential impacts of elevated IOP on RGC dendrite structure, this could be mediated by a loss of specific intraretinal synaptic contacts.^31,36,51^ Future studies will be required to distinguish from among these possibilities.
